# Hierarchical Spatial Concept Formation Based on Multimodal Information for Human Support Robots

**DOI:** 10.3389/fnbot.2018.00011

**Published:** 2018-03-13

**Authors:** Yoshinobu Hagiwara, Masakazu Inoue, Hiroyoshi Kobayashi, Tadahiro Taniguchi

**Affiliations:** Emergent Systems Laboratory, College of Information Science and Engineering, Ritsumeikan University, Shiga, Japan

**Keywords:** spatial concept, hierarchy, human-robot interaction, multimodal categorization, human support robot, unsupervised learning

## Abstract

In this paper, we propose a hierarchical spatial concept formation method based on the Bayesian generative model with multimodal information e.g., vision, position and word information. Since humans have the ability to select an appropriate level of abstraction according to the situation and describe their position linguistically, e.g., “I am in my home” and “I am in front of the table,” a hierarchical structure of spatial concepts is necessary in order for human support robots to communicate smoothly with users. The proposed method enables a robot to form hierarchical spatial concepts by categorizing multimodal information using hierarchical multimodal latent Dirichlet allocation (hMLDA). Object recognition results using convolutional neural network (CNN), hierarchical k-means clustering result of self-position estimated by Monte Carlo localization (MCL), and a set of location names are used, respectively, as features in vision, position, and word information. Experiments in forming hierarchical spatial concepts and evaluating how the proposed method can predict unobserved location names and position categories are performed using a robot in the real world. Results verify that, relative to comparable baseline methods, the proposed method enables a robot to predict location names and position categories closer to predictions made by humans. As an application example of the proposed method in a home environment, a demonstration in which a human support robot moves to an instructed place based on human speech instructions is achieved based on the formed hierarchical spatial concept.

## 1. Introduction

Space categorization is an important function for human support robots. It is believed that humans predict unknown information flexibly by forming categories of space through their multimodal experiences. We define categories of spaces formed by self-organization from experience as spatial concepts. Furthermore, prediction based on the connection between concepts and words is thought to lead to a semantic understanding of words. It means that spatial concept formation is an important function of human intelligence, and having this ability is important for human support robots.

Spatial concepts form a hierarchical structure. The use of this hierarchical structure enables humans to predict unknown information using concepts in an appropriate layer. For example, humans can linguistically represent their own positions at an appropriate level of abstraction according to the context of communication, such as “I'm in my home” at the global level, “I'm in the living room” at the intermediate level, and “I'm in front of the TV” at the local level. In this case, the living room has the home in the higher layer and front of the TV in the lower layer. By learning such a hierarchical structure, even if the unknown place does not have features such as front of the TV, its characteristics can be predicted if it has features of the living room. It is expected that the robot acquires spatial concepts in a higher layer by learning the commonality of features in spatial concepts at the lower layer.

**Figure 1:**
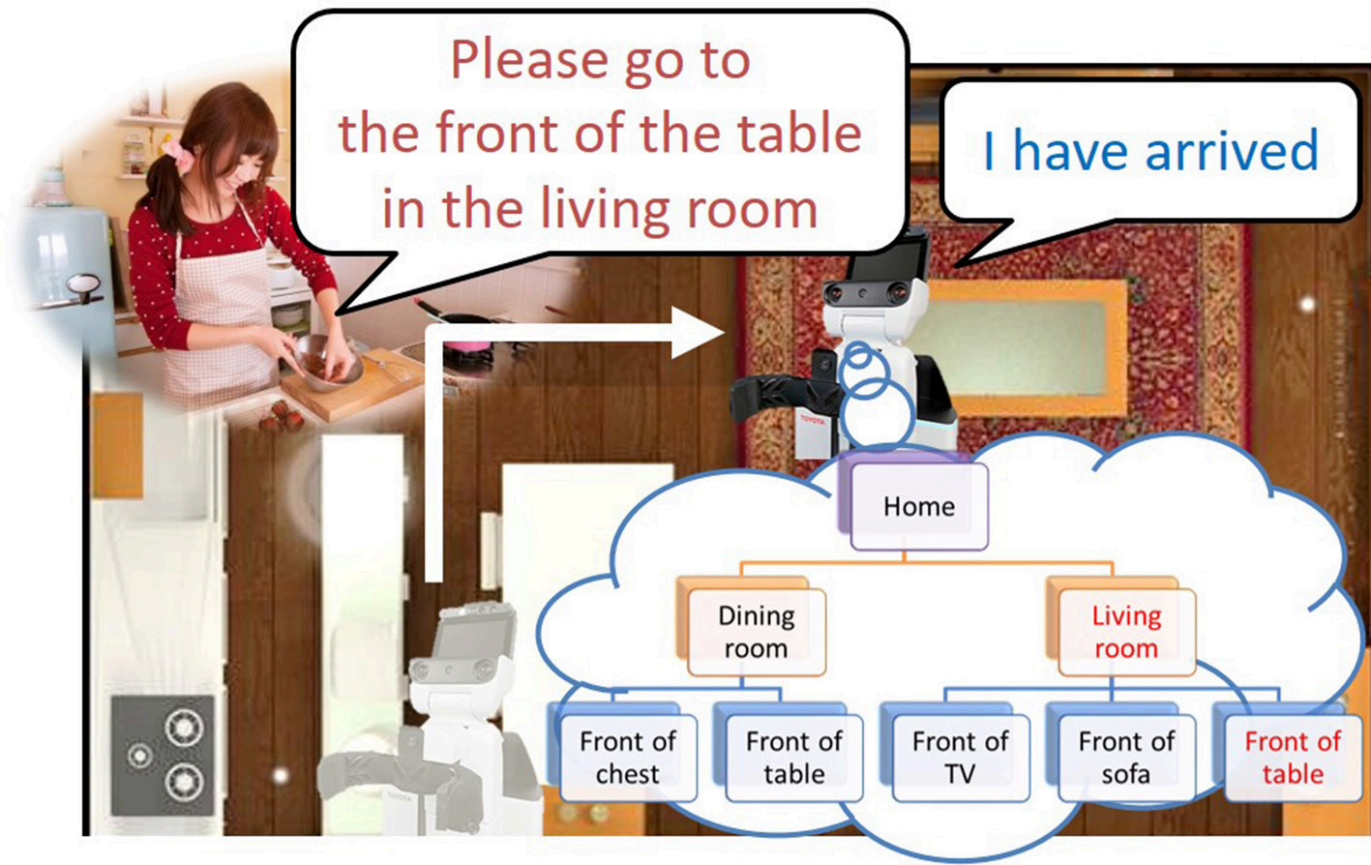
Example of movement based on linguistic instructions with a hierarchical space structure.

Furthermore, the hierarchical structure of spatial concepts plays an important role when a robot moves based on linguistic instructions from a user. As shown in Figure 1, even if multiple tables are present in a room, robots can recognize them individually by using a spatial concept at a higher layer, such as “the front of the table in the living space.” Indeed, in RoboCup@Home, an international competition in which intelligent robots coexist with humans in home environments, location names are defined as two layers in the tasks of a General Purpose Service Robot^1^ as shown in Table 1. This table indicates that having sense of space relations is important for a robot coexisting with humans, e.g., that the living space has a center table. By having such hierarchical spatial concepts, it becomes possible to describe and move within a space based on linguistic communication with a user.

**Table 1:** Definition of location names with two layers in RoboCup@Home.

**Name (1st layer)**	**Name (2nd layer)**
Living room	Bar
Living room	TV stand
Living room	Center table
Office	Drawer
Office	Desk
Kitchen	Bar
Kitchen	Cupboard
Bathroom	Cupboard

We assume that a computational model, which considers the hierarchical structure of spatial concepts, enables robots to acquire not only the spatial concepts, but also the hierarchical structure hiding among the spatial concepts through a bottom-up approach and form spatial concepts similar to those perceived by humans. The goal of this study was to develop a robot that can predict unobserved location names and positions from observed information using formed hierarchical spatial concepts. The main contributions of this paper are as follows.

- We propose a method of forming hierarchical spatial concepts using a Bayesian generative model based on multimodal information, e.g., vision, position, and word information.
- We show that spatial concepts formed by the proposed method enable a robot to predict location names and positions similar to prediction made by humans.
- We demonstrate application examples in which a robot moves within and describes a space based on linguistic communication with a user through the hierarchical spatial concept formed by the proposed method.

The rest of this paper is structured as follows. Section 2 describes related works. Section 3 presents an overview and the computational model of hierarchical spatial concept formation. Section 4 presents experimental results evaluating the effectiveness of the proposed method in space categorization. Section 5 describes application examples of using hierarchical spatial concepts in a home environment. Finally, section 6 presents conclusions.

## 2. Related works

In order for a robot to move within a space, a metric map consisting of occupancy grids that encode whether or not an area is navigable is generally used. The simultaneous localization and mapping (SLAM) (Durrant-Whyte and Bailey, 2006) is a famous localization method for mobile robots. However, the tasks that are coordinated with a user cannot be performed using only a metric map, since semantic information is required for interaction with a user. Nielsen et al. (2004) proposed a method of expanding a metric map into a semantic map by attaching a single-frame snapshot in order to share spatial information between a user and a robot. As a bridge between a metric map and human-robot interaction, research on semantic maps that provide semantic attributes (such as object recognition results) to metric maps has been performed (Pronobis et al., 2006; Ranganathan and Dellaert, 2007). Studies have also been reported on giving semantic object annotations to 3D point cloud data (Rusu et al., 2008, 2009). Moreover, in terms of studies based on multiple cues, Espinace et al. (2013) proposed a method of characterizing places according to low-level visual features associated to objects. Although these approaches could categorize spaces based on semantic information, they did not deal with linguistic information about the names that represent spaces.

In the field of navigation tasks with human-robot interaction, methods of classifying corridors and rooms using a predefined ontology based on shape and image features have been proposed (Zender et al., 2008; Pronobis and Jensfelt, 2012). In studies on semantic space categorization, Kostavelis and Gasteratos (2013) proposed a method of generating a 3D metric map that is semantically categorized by recognizing a place using bag of features and support vector machines. Granda et al. (2010) performed spatial labeling and region segmentation by applying a Gaussian model to the SLAM module of a robot operating system (ROS). Mozos and Burgard (2006) proposed a method of classifying metric maps into semantic classes by using adaboost as a supervised learning method. Galindo et al. (2008) utilized semantic maps and predefined hierarchical spatial information for robot task planning. Although these approaches were able to ground several predefined names to spaces, the learning of location names through human-robot communication in a bottom-up manner has not been achieved.

Many studies have been conducted on spatial concept formation based on multimodal information observed in individual environments (Hagiwara et al., 2016; Heath et al., 2016; Rangel et al., 2017). Spatial concepts are formed in a bottom-up manner based on multimodal observed information, and allow predictions of different modalities. This makes it possible to estimate the linguistic information representing a space from position and image information in a probabilistic way. Gu et al. (2016) proposed a method of learning relative space categories from ambiguous instructions. Taniguchi et al. (2014, 2016) proposed computational models for a mobile robot to acquire spatial concepts based on information from recognized speech and estimated self-location. Here, the spatial concept was defined as the distributions of names and positions at each place. The method enables a robot to predict a positional distribution from recognized human speech through formed spatial concepts. Ishibushi et al. (2015) proposed a method of learning the spatial regions at each place by stochastically integrating image recognition results and estimated self-positions. In these studies, it was possible to form a spatial concept conforming to human perception such as an entrance and a corridor by inferring the parameters of the model.

However, these studies did not focus on the hierarchical structure of spatial concepts. In particular, the features of the higher layer, such as the living space, are included in the features of the lower layer, such as the front of the television, and it was difficult to form the spatial concept in the abstract layer. Furthermore, the ability to understand and describe a place linguistically in different layers is an important function in robots that provide services through linguistic communication with humans. Despite the importance of the hierarchical structure of spatial concepts, a method that enables such concept formation has not been proposed in previous studies. We propose a method that forms a hierarchical spatial concept in a bottom-up manner from multimodal information and demonstrate the effectiveness of the formed spatial concepts in predicting location names and positions.

## 3. Hierarchical space concept formation method

### 3.1. Overview

**Figure 2:**
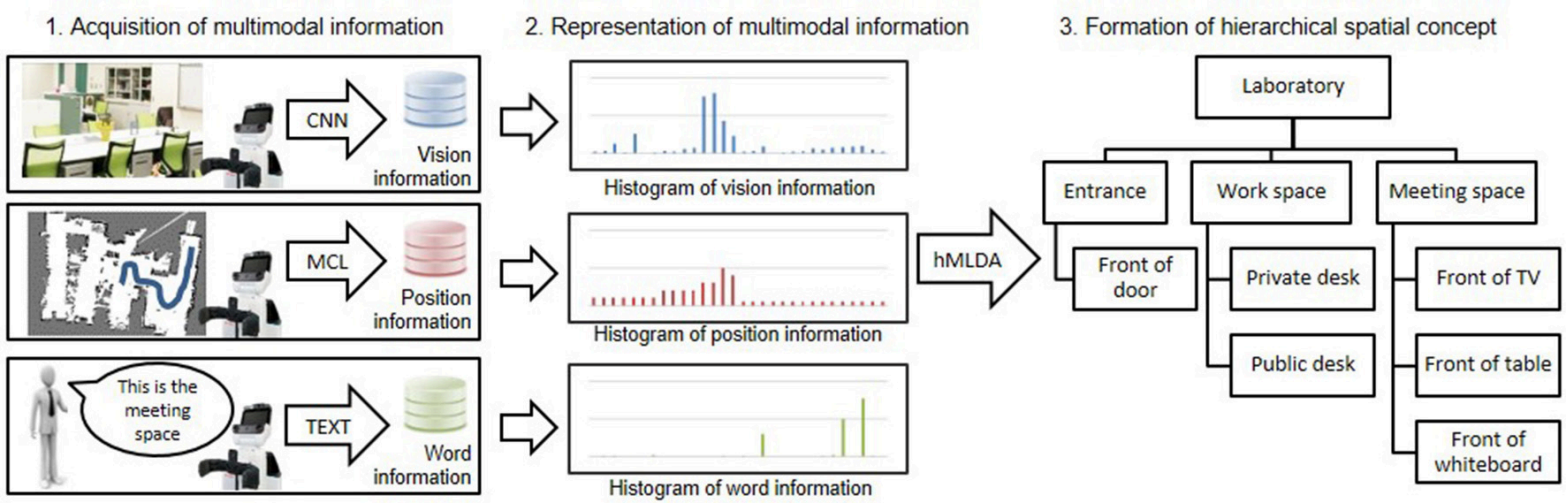
Overview of the proposed method for hierarchical spatial concept formation.

An overview of the proposed method of forming hierarchical spatial concepts is shown in Figure 2. First, a robot was controlled manually in an environment based on a map generated by simultaneous localization and mapping (SLAM) (Durrant-Whyte and Bailey, 2006) and acquires multimodal information, i.e., vision, position, and word information from attached sensors. Vision information is acquired as a feature vector generated by a convolutional neural network (CNN) (Krizhevsky et al., 2012). Position information is acquired as coordinate values in the map estimated by Monte Carlo localization (MCL) (Dellaert et al., 1999). Word information is acquired as set of words by word recognition. Text input is used for word recognition in this study. Second, acquired vision, position, and word information is represented as histograms. The histograms are utilized as observations in each modality. Third, the formation of hierarchical spatial concepts is performed by using hierarchical multimodal latent Dirichlet allocation (hMLDA) (Ando et al., 2013) on the observations. The proposed method enables a robot to form hierarchical spatial concepts in a bottom-up manner based on observed multimodal information. Therefore, it is possible to adaptively learn location names and the hierarchical structure of a space, which depend on the environment.

### 3.2. Acquisition and feature extraction of multimodal information

#### 3.2.1. Vision information

Vision information was acquired as the object recognition results of a captured image by Caffe (Jia et al., 2014), which is a framework of CNN (Krizhevsky et al., 2012) provided by Berkeley Vision and Learning Center. The parameters of CNN were trained by using the dataset from the ImageNet Large Scale Visual Recognition Challenge 2013^2^, which comprises 1,000 object classes, e.g., television, cup, and desk. The output of Caffe is given as a probability *p*(*a*_*i*_) at an object class *a*_*i*_ ∈ {*a*_1_, *a*_2_, …, *a*_*I*_} where *I* is the number of object classes and was set to 1,000. The probability *p*(*a*_*i*_) was represented as a 1,000-dimensional histogram of vision information $w^{\left(u\right)}=(w_{1}^{\left(\upsilon \right)},w_{2}^{\left(\upsilon \right)},\ldots ,w_{1,000}^{\left(\upsilon \right)}{)}^{T}$ by the following equation:

(1) $$\begin{array}{l} w_{i}^{\left(\upsilon \right)}=p\left(a_{i}\right)\;*\;10^{2}. \end{array}$$

#### 3.2.2. Position information

The position information (*x, y*) in the map generated by SLAM was estimated by MCL (Dellaert et al., 1999). It is assumed that the observed information is generated from a multinomial distribution in hMLDA. For this reason, the observed information with a continuous value is generally converted into a finite dimensional histogram by vector quantization. Ando et al. (2013) replaced the observed information with typical patterns by k-means clustering to form a finite dimensional histogram. The proposed method converts a position information (*x, y*) into a finite dimensional histogram of position information ***w***^*p*^ by hierarchical k-means clustering. The positional information (*x, y*) was classified hierarchically into 2, 4, 8, 16, 32, and 64 clusters with six layers by applying k-means clustering with *k* = 2 six times. If a position (*x, y*) was classified into a cluster *c*_*i*_ ∈ {0, 1} at the *i*th layer, a path for the position information was described as *C* = {*c*_1_, *c*_2_, *c*_3_, *c*_4_, *c*_5_, *c*_6_}. The path *C* has the structure of a binary tree with six layers. The number of nodes at the 6th layer is 2^6^ = 64. The position information (*x, y*) is represented as a 64-dimensional histogram of the position information $w^{\left(p\right)}=(w_{1}^{\left(p\right)},w_{2}^{\left(p\right)},\ldots ,w_{64}^{\left(p\right)}{)}^{T}$ by incrementing $w_{i}^{\left(p\right)}$ based on the path *C*. For example, in a path *C* of position information (*x, y*), when *c*_1_ = 0, $w_{1}^{\left(p\right)}$ to $w_{32}^{\left(p\right)}$ corresponding to nodes at the 6th layer are incremented, and when *c*_1_ = 1, $w_{33}^{\left(p\right)}$ to $w_{64}^{\left(p\right)}$ are incremented. Similarly, *w*^(*p*)^ corresponding to nodes at the 6th layer below it are incremented in each layer.

#### 3.2.3. Word information

The voice information uttered by a user is converted manually into text data, which is then used as word information. In section 5, rospeex (Sugiura and Zettsu, 2015) is used to convert human speech into text data. The location names are manually extracted from the text data. The word information is described as a set of location names, which is a Bag of Words (Harris, 1954) with a location name as a word. The user could give not only one name but also several names to a robot at a given position. The given word information was represented as a histogram of word information $w^{\left(w\right)}=(w_{1}^{\left(w\right)},w_{2}^{\left(w\right)},\ldots ,w_{J}^{\left(w\right)})$^*T*^. *J* is the dimension of ***w***^(*w*)^, and depends on the number of location names in a dictionary *S* = {*s*_1_, *s*_2_,…,*s*_*J*_}, which was obtained through the training phase. $w_{j}^{\left(w\right)}$ was incremented when a location name *s*_*j*_ was taught from user. *J* is the number of location names. Histograms of vision, position, and word information were used as observations in hMLDA.

### 3.3. Hierarchical categorization by hMLDA

**Figure 3:**
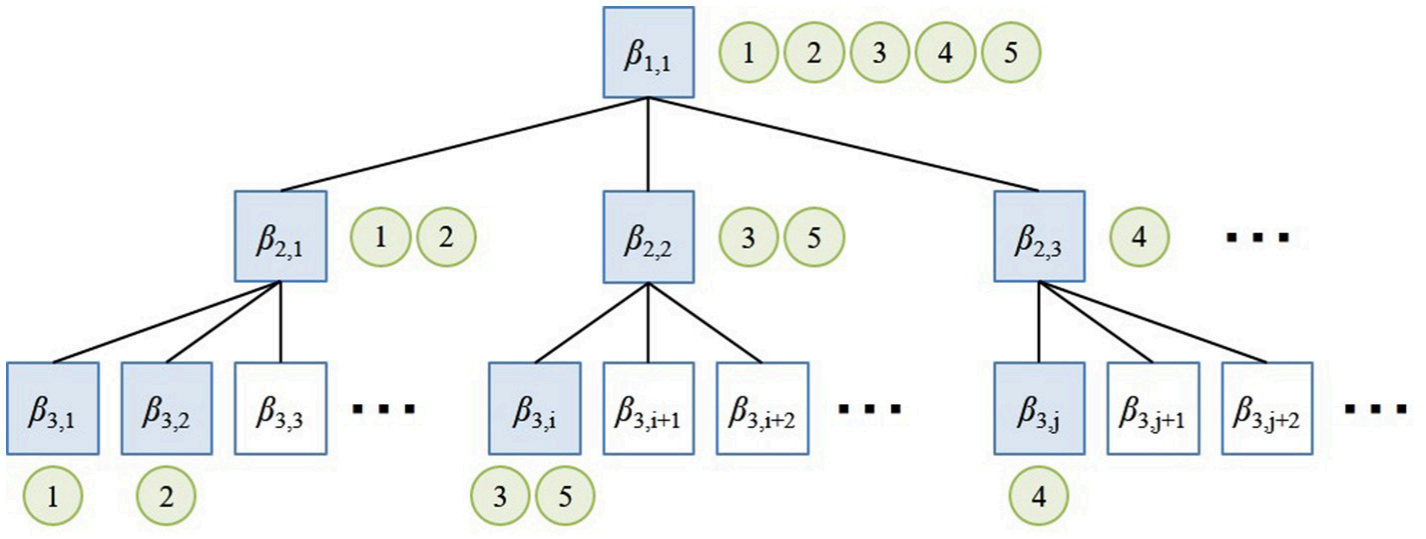
Overview of nested Chinese restaurant process (nCRP).

The hierarchical structure of spatial concepts is supported by nested Chinese restaurant process (nCRP) (Blei et al., 2010) in hMLDA (Ando et al., 2013). nCRP is an extended model of the Chinese restaurant process (CRP) (Aldous, 1985), which is a Dirichlet process used to generate multinomial distribution with infinite dimensions. nCRP stochastically calculates the hierarchical structure based on the idea that there are infinite Chinese restaurants with infinite number of tables. Figure 3 shows the overview of nCRP. A box and a circle represent a restaurant and a customer, respectively. The customer stochastically decides the restaurant to visit. In the proposed method, a box and a circle mean a spatial concept and data, respectively. Data is stochastically allocated to a spatial concept in each layer by the nCRP. In hMLDA, each spatial concept has a probability distribution with parameter β_*l,i*_ to generate data. The proposed method forms a hierarchical spatial concept by hierarchical probabilistic categorization using nCRP. In the non-hierarchical approach, a place called “meeting space” and its partial places called “front of the table” and “front of the TV” are formed in the same layer. Therefore, the meeting space is learned as a place different from places called “front of the table” and “front of the TV.” The proposed method enables the robot to learn the meeting space as a upper concept encompassing places called “front of the table” and “front of the TV” as lower concepts.

**Figure 4:**
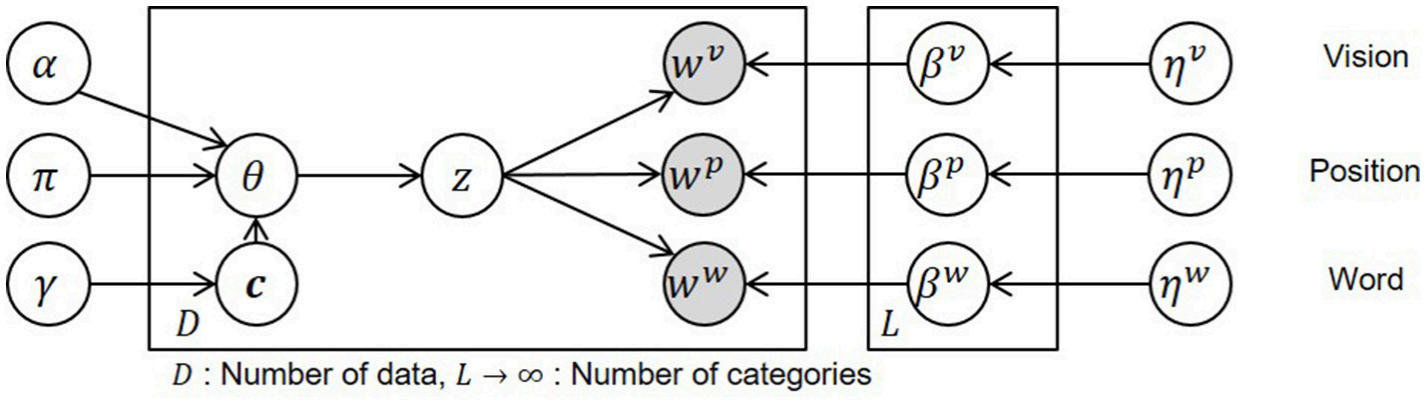
Graphical model of hierarchical spatial concept formation.

The graphical model of hMLDA in the proposed method and the definition of the variables are shown in Figure 4 and Table 2, respectively. In Figure 4, ***c*** is a tree-structured path generated by nCRP with a parameter γ and *z* is a category index for a spatial concept that is generated by a stick-breaking process (Pitman, 2002) with parameters α and π. *w*^υ^, *w*^*p*^, *w*^*w*^ are acquired vision, position, and word information generated by multinomial distributions with a parameter β^*m*^ at a modality *m* (*m* ∈ υ, *p, w*). β^*m*^ was determined according to a Dirichlet prior distribution with a parameter η^*m*^. *D* and *L* written on plates are the number of acquired data and the number of categories, respectively.

**Table 2:** Definition of variables in the graphical model.

**Variable**	**Definition**
*w*^υ^, *w*^*p*^, *w*^*n*^	Observation of vision, position and word information
*z*	Index of category
β^υ^, β^*p*^, β^*n*^	Parameter of multinomial distribution in vision, position and word information
θ	Parameter of multinomial distribution in category
**c**	Path of tree structure
η^υ^, η^υ^, η^*w*^	Parameter of Dirichlet prior distribution
γ	Hyper parameter of **c**
α, π	Hyper parameter of θ

The generation process of the model is described as follows:

(2) $$\begin{array}{l} \beta_{k}^{m}\sim \mathrm{Dirichlet}\left(\eta^{m}\right) \end{array}$$

(3) $$\begin{array}{l} {\text{c}}_{d}\sim \mathrm{nCRP}\left(\gamma \right) \end{array}$$

(4) $$\begin{array}{l} \theta_{d}\sim \mathrm{GEM}\left(\alpha ,\pi \right) \end{array}$$

(5) $$\begin{array}{l} z_{d,n}^{m}\sim \mathrm{Multi}\left(\theta_{d}\right) \end{array}$$

(6) $$\begin{array}{l} w_{d}^{m}\sim \mathrm{Multi}\left(\beta_{{\text{c}}_{d}}\left[z_{d,n}^{m}\right]\right), \end{array}$$

where:

- The parameter $\beta_{k}^{m}$ of a multinomial distribution is generated by a Dirichlet prior distribution with a parameter η^*m*^ in a table *k*(*k* ∈ *T*), e.g., β_1,1_ and β_2,1_ in Figure 3.
- The path *c*_*d*_ in a tree structure for the data *d* (*d* ∈ 1, 2, …, *D*) is decided by nCRP with a parameter γ. *c*_*d*_ is represented by the sequence of numbers assigned to each node in the path, e.g., {(1, 1), (2, 1), (3, 2)} at data 2 in Figure 3.
- The parameter θ_*d*_ of a multinomial distribution is generated by the stick-breaking process based on a GEM distribution which forms θ_*d*_ from a Beta(απ, (1 − α)π) distribution with the parameters α(0 ≤ α ≤ 1) and π(π > 0) (Pitman, 2006). θ_*d*_ represents the selection probability of a layer in a path *c*_*d*_ and corresponds to the generation probability of a category index *z* in a path *c*_*d*_.
- $z_{d,n}^{m}$, which is a category index at the *n*th feature of the observed information $w_{d}^{m}$, is generated by a multinomial distribution with a parameter θ_*d*_.
- $w_{d}^{m}$ is the observed information generated by a multinomial distribution with a parameter β from a category $z_{d,n}^{m}$ at a path *c*_*d*_.

In this study, *z* is equivalent to a spatial concept expressed by the location name such as “the living room” or “front of the table.”

Model parameter learning was performed by a Gibbs sampler. Parameters were calculated by alternately sampling a path *c*_*d*_ for each datum and a category $z_{d,n}^{m}$ assigned to the *n*th feature value of a modality *m* of the data *d* in the path. Category $z_{d,n}^{m}$ was sampled according to the following formula.

(7) $$\begin{array}{l} z_{d,n}^{m}\sim p(z_{d,n}^{m}|z_{-(d,n)}^{m},c,w^{m},\alpha ,\pi ,\eta^{m})\\ \;\propto p(z_{d,n}^{m},z_{-(d,n)}^{m},c,w^{m}|\alpha ,\pi ,\eta )\\ \;\propto p(z_{d,n}^{m}|z_{d,-n}^{m},\alpha ,\pi )p(w_{d,n}^{m}|z,c,w_{-(d,n)}^{m},\eta^{m}), \end{array}$$

where −(*d, n*) means excluding the *n*th feature value of the data *d*. $p\left(z_{d,n}^{m}|{\text{z}}_{d,-n}^{m},\alpha ,\pi \right)$ is a multinomial distribution generated by the stick-breaking process. The probability, that *k* is assigned to a category of the *n*-th feature of modality *m* of the *d*-th data, was calculated by the following formula.

(8) $$\begin{array}{l} p(z_{d,n}^{m}=k|z_{d,-n}^{m},\alpha ,\pi )=E[V_{k}\prod_{j=1}^{k-1}\left(1-V_{j}\right)|z_{d,-n}^{m},\alpha ,\pi ]\\ \;=E[V_{k}|z_{d,-n}^{m},\alpha ,\pi ]\prod_{j=1}^{k-1}E[1-V_{j}|z_{d,-n}^{m},\alpha ,\pi ]\\ \;=\frac{\left(1-\alpha )\pi +\#[z_{d,-n}^{m}=k\right]}{\pi +\#[z_{d,-n}^{m}\geq k]}\prod_{j=1}^{k-1}\frac{\alpha \pi +\#[z_{d,-n}^{m}>j]}{\pi +\#[z_{d,-n}^{m}\geq j]}, \end{array}$$

where #[·] is a number that satisfies a given condition and *V*_*k*_ and *V*_*j*_ are values that determine the rate of folding a branch in categories *k* and *j* by the stick-breaking process, respectively.

In Formula (7), $p\left(w_{d,n}^{m}|\text{z},\text{c},{\text{w}}_{-\left(d,n\right)}^{m},\eta^{m}\right)$ is the probability that a feature value is generated from a path **c**_*d*_ and a category $z_{d,n}^{m}$. Since it is assumed that the parameters of the multinomial distribution that generates a feature value are generated from a Dirichlet prior distribution, the following formula is obtained.

(9) $$\begin{array}{l} p(w_{d,n}^{m}|z,c,w_{d,n}^{m},\eta^{m})\propto \#[z_{-(d,n)}^{m}=z_{d,n}^{m},c_{z_{d,n}^{m}}=c_{d,z_{d,n}^{m}},w_{-(d,n)}^{m}\\ \;=w_{d,n}^{m}]+\eta^{m} \end{array}$$

This gives the number of times that a category $z_{d,n}^{m}$ is assigned to a feature value $w_{d,n}^{m}$ in a path **c**_*d*_. A path *c*_*d*_ was sampled by the following formula.

(10) $$\begin{array}{l} c_{d}\sim p(c_{d}|w^{v},w^{p},w^{w},c_{-d},z,\eta^{v},\eta^{p},\eta^{w},\gamma )\\ \;\propto p(c_{d}|c_{-d},\gamma )p(w_{d}^{v}|c,w_{-d}^{v},z^{v},\eta^{v})p(w_{d}^{p}|c,w_{-d}^{p},z^{p},\eta^{p})\\ \;p(w_{d}^{w}|c,w_{-d}^{w},z^{w},\eta^{w}), \end{array}$$

where **c**_−*d*_ is a set of paths excluding **c** from **c**_*d*_. Sampling based on Formulas (9) and (10) was repeated for each training datum *d* ∈ {*d*_1_, *d*_2_, …, *d*_*D*_}. In this process, paths and categories for all observed data converge to **ĉ** and **ẑ**.

### 3.4. Name prediction and position category prediction

If vision information $w_{t}^{v}$ and position information ${\text{w}}_{t}^{p}$ are observed at a time *t*, then the posterior probability of word information $w_{t}^{w}$ can be calculated with estimated parameters **ĉ** and **ẑ** by the following formula.

(11) $$\begin{array}{l} p(w_{t}^{w}|\hat{z},\hat{c},w^{w},w^{v},w^{p},c_{t},w_{t}^{v},w_{t}^{p},\alpha ,\pi ,\eta^{n},\eta^{v},\eta^{p})=\\ \;\sum_{z_{t}}p(w_{t}^{w}|z_{t},{\hat{z}}^{w},\hat{c},w^{w},\eta^{w})\\ \;p(z_{t}|{\hat{z}}^{v},{\hat{z}}^{p},\hat{c},w^{v},w^{p},c_{t},w_{t}^{v},w_{t}^{p},\alpha ,\pi ,\eta^{v},\eta^{p}) \end{array}$$

The location name $\hat{n}$ can be predicted by the maximum value of the calculated posterior probability.

If word information $w_{t}^{w}$ is obtained at a time *t*, then a category $z_{t}^{w}$ can be predicted by Formula (12) and selecting position $\hat{p}$ randomly from dataset $D_{z_{t}^{w}}$, which is a set of position data categorized into $z_{t}^{w}$. $D_{z_{t}^{w}}$ was automatically generated by the robot itself as a part of the categorization process.

(12) $$z_{t}^{w}\sim p(z_{t}^{w}|z_{-t}^{w},w_{t}^{w},\hat{c},w^{w},w^{v},w^{p},\eta^{w},\eta^{v},\eta^{p},\alpha ,\pi )$$

## 4. Experiment

### 4.1. Purpose

We conducted experiments to verify whether the proposed method can form hierarchical spatial concepts, which enable a robot to predict location names and position categories close to predictions made by humans. In the experiment, (1) the influence of multimodal information, i.e., words, on the formation of a hierarchical spatial concept was evaluated by comparing the space categorization results of using the proposed method and those of hierarchical latent Dirichlet allocation (hLDA) (Blei et al., 2010), which is a hierarchical categorization method with single modality; (2) the similarity between the hierarchical spatial concepts formed by the proposed method and those made by humans was evaluated in terms of predicting location names and position categories.

### 4.2. Experimental conditions

**Figure 5:**
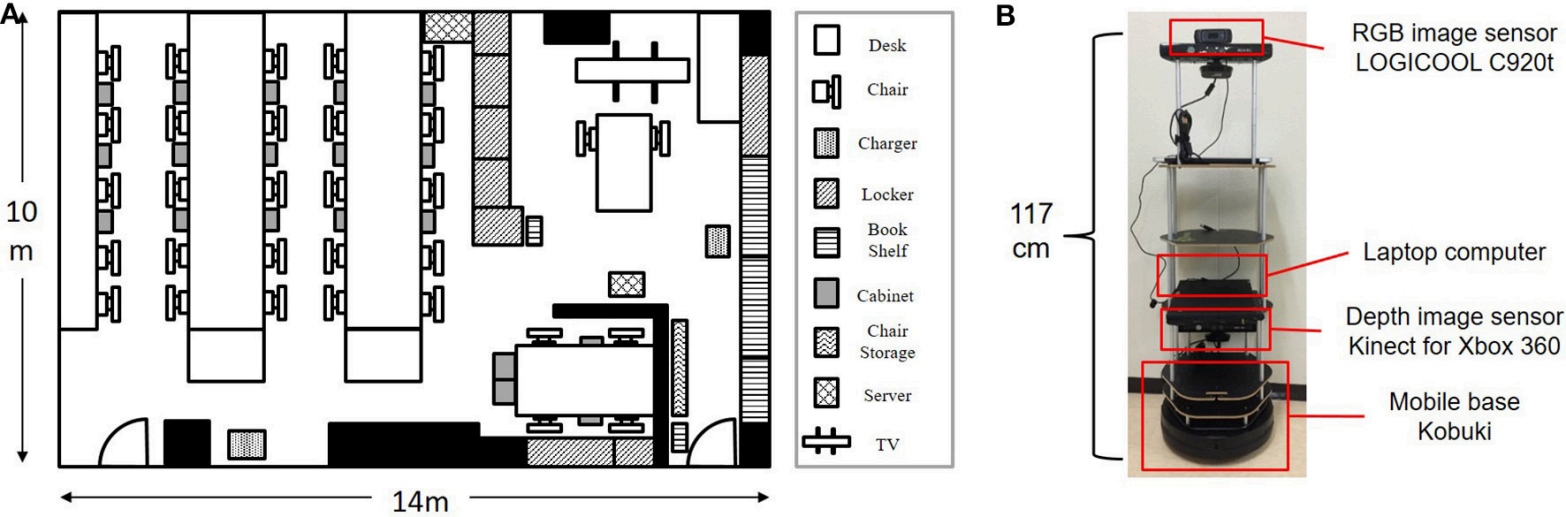
Experimental environment and mobile robot for learning and testing. **(A)** Experimental environment for data collection. **(B)** Mobile robot used for experiments.

Figure 5A shows an experimental environment which includes furniture, e.g., tables, chairs, and a book shelf, in order to collect training and test data. Figure 5B shows a mobile robot, which consists of a mobile base, a depth sensor, an image sensor, and a computer, used to generate a map and collect multimodal information in the test environment. The height of the camera attached to the robot was 117 cm in consideration of the typical eye level in the human body. This is equivalent to the average height of a 5-year-old boy in Japan. The Navigation Stack package^3^ was used with ROS Hydro^4^ for mapping, localization, and moving in the experiment. The robot was manually controlled to collect data from the environment. The orientation of the robot was controlled in as many different orientations as possible.

**Figure 6:**
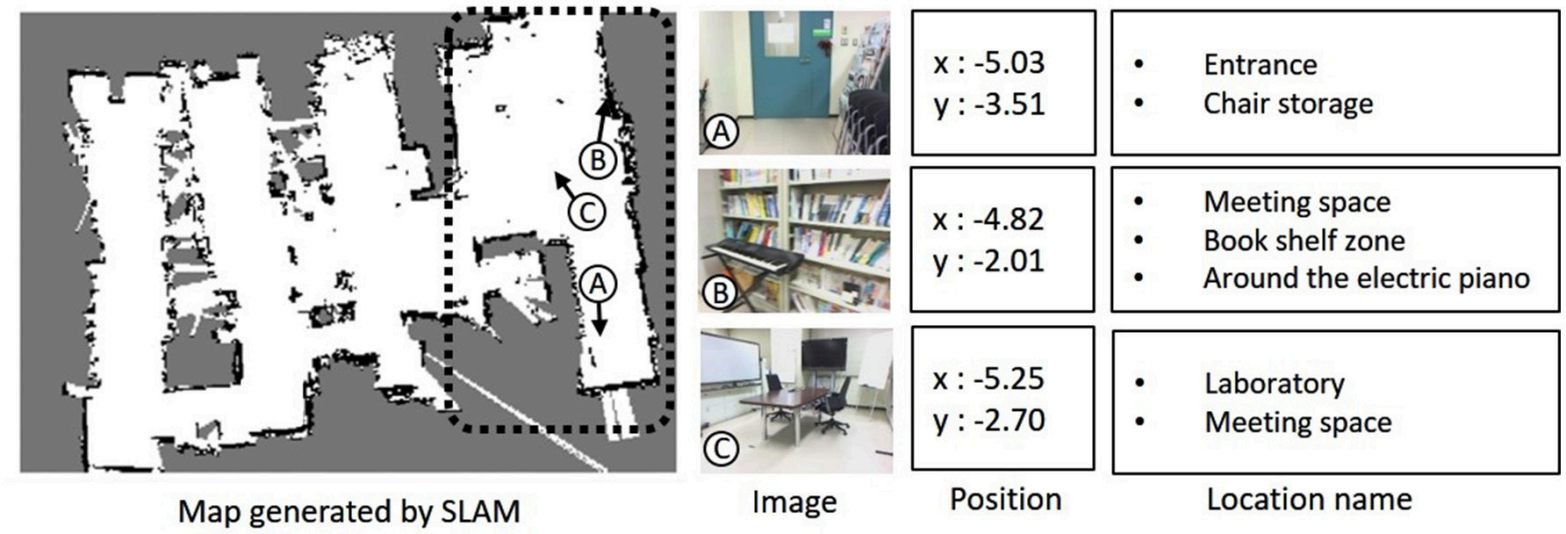
Map generated by SLAM and examples of collected data: image, position, and location name.

Figure 6 shows a map generated in the environment by the robot using SLAM and examples of the collected data. Collected data consisted of image, position, and word information as shown in the samples of collected data at A, B, and C. In the experiment, 900 data points were used for training and 100 data points were used for testing from a total of 1,000 data points collected in the area surrounded by a dotted line in the map. The robot simultaneously acquired images and self-position data (*x, y*) at times of particle re-sampling for MCL. Words were given as location names by a user who was familiar with the experimental environment. The user gave one or more location names suitable for the place at a data point during the training. In example A, not only a name such as “front of the door” but also a name representing a space such as “entrance” and a name meaning a room such as “laboratory” were given as word information. Word information was partially supplied as training data. Five training data sets were prepared to evaluate robustness of the naming rate in training data as 1, 2, 5, 10, and 20%.

**Figure 7:**
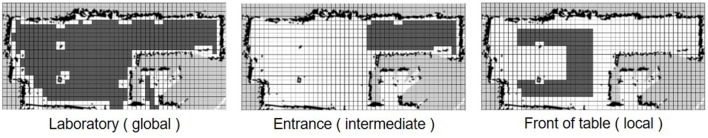
Examples of ground truth for regions where the location names are at the global, intermediate, and local levels. The area is mapped by a grid of 50 columns and 25 rows. The region of ground truth is represented by the gray grids.

The similarity between the spatial concepts formed by the proposed method and those made by humans was evaluated in experiments of location name prediction and position category prediction based on the ground truth. The ground truth information was given for 100 test data points according to the agreement of three experts who were familiar with the environment. The hierarchy of the space in the experimental environment was defined as global, intermediate, and local. Location names assigned to each hierarchy are shown in Table 3. As the ground truth for name prediction, three location names were uniformly given to each test datum considering the hierarchy to evaluate the accuracy of name prediction. As the ground truth for the position category prediction, regions corresponding to the 15 location names in Table 3 were decided on the map. Figure 7 shows the three regions of the “laboratory,” “entrance,” and “front of the table.” The environment was divided into a grid of 50 units in length and 25 units in width, and the gray grids show the ground truth.

**Table 3:** List of location names and ground truth in the hierarchy.

**Global**	Laboratory		
**Intermediate**	Entrance	Meeting space	
**Local**	Front of the door	Umbrella storage	Magazine rack zone
	Chair storage	Book shelf zone	Around Skype PC
	Around the charger	Around the electric piano	Locker zone
	Front of the white board	Front of the display	Front of the table

In the name prediction experiment, the accuracy of name prediction compared with the ground truth was calculated as an index of similarity. Formula (11) was used to predict names using the proposed method. The accuracy of name prediction at global, intermediate, and local levels was calculated by the following formula.

(13) $$\begin{array}{l} Accuracy=\frac{M_{l}}{D}, \end{array}$$

where *M*_*l*_ is a number matching the predicted names with the ground truth at layer *l* in the test dataset and *D* is the number of test data. In the experiment, *l* was set as (*l* ∈ {*global, intermediate, local*}) and *D* was 100.

In the position category prediction experiment, the precision, recall, and F-measure of the predicted position categories compared with the ground truth were calculated as an index of similarity. In the proposed method, a position (*x, y*) sampled multiple times for each location name by Formula (12). The precision, recall, and F-measure of position category prediction were calculated by the following formulas.

(14) $$\begin{array}{l} Precision=\frac{T_{n}}{T_{n}+F_{n}} \end{array}$$

(15) $$\begin{array}{l} Recall=\frac{T_{n}}{G_{n}} \end{array}$$

(16) $$\begin{array}{l} F-measure=\frac{2\cdot Recall\cdot Precision}{Recall+Precision}, \end{array}$$

where *T*_*n*_ is a number matching the position with the ground truth for location name *n*, *F*_*n*_ is a number that does not match the position with the ground truth, and *G*_*n*_ is the number of grids for the ground truth. In the experiment, *n* was set as (*n* ∈ {1, 2, …, 15}).

In the proposed method, the hyper-parameters α, π, γ, η were set as α = 0.5, π = 100, γ = 1.0, η^υ^ = 1.0 × 10^−1^, η^*p*^ = 1.0 × 10^−3^, η^*w*^ = 1.0 × 10^−2^, respectively. The path *c* and category *z* of each data were trained with the hyper-parameters. In the experiment, the dimensions of the information vectors *w*^υ^, *w*^*p*^, and *w*^*w*^ were 1,000, 64, and 15, respectively.

### 4.3. Baseline methods

The most frequent class, nearest neighbor method, multimodal hierarchical Dirichlet process (HDP), and spatial concept formation model were used as baseline methods for evaluating the performance of the proposed method in the name prediction and position category prediction experiments. In the latter, the sampling of position for each location name was performed 100 times.

#### 4.3.1. Most frequent class

The training dataset *D* = {*d*_1_, *d*_2_, …, *d*_*I*_} is used in this method. The datum *d*_*i*_ consists of the position information *p*_*i*_ = (*x*_*i*_, *y*_*i*_) and the word information *w*_*i*_, which is a set of location names. The frequency *cnt*_*n*_*j*__ of each location name *n*_*j*_(*j* ∈ {1, 2, …, 15}) is counted in the training dataset *D*. The location name *n*_*j*_ is classified into three clusters by k-means (*k* = 3) based on *cnt*_*n*_*j*__. The three clusters of location names are *C*_*global*_, *C*_*intermediate*_, and *C*_*local*_ in descending order of the frequency of the location name based on the assumption that global location names are more frequent than local location names. In the training dataset *D*, if a datum *d*_*i*_ includes a location name in *C*_*global*_, *C*_*intermediate*_, and *C*_*local*_, the datum *d*_*i*_ is set as a global dataset *D*_*g*_, an intermediate dataset *D*_*i*_, and a local dataset *D*_*l*_. The location names in the global, intermediate, and local levels are predicted as the most frequent location name in each dataset *D*_*g*_, *D*_*i*_, and *D*_*l*_, respectively.

In the position category prediction, the positions are predicted by sampling the position information $\hat{p}$ randomly from the datasets *D*_*g,f*_, *D*_*i,f*_, and *D*_*l,f*_, which have the most frequent location names in each dataset *D*_*g*_, *D*_*i*_, and *D*_*l*_, respectively. The sampling of position information for each location name was performed 100 times.

#### 4.3.2. Nearest neighbor (position and word)

The nearest neighbor method (Friedman et al., 1977) discriminates data based on Euclidean distance. A datum *d*_*i*_ involves position information *p*_*i*_ = (*x*_*i*_, *y*_*i*_) and word information *w*_*i*_. *w*_*i*_ consists of a set of location names that obtained at a position *p*_*i*_ in the training. For example, *w*_*i*_ at data point B in Figure 6 contains the following location names: “Meeting space,” “Book shelf zone,” and “Around the electric piano.” If position information *p*_*t*_ is observed, then word information $\hat{w_{t}}$ is calculated with the training dataset *D* = {(*p*_1_, *w*_1_), (*p*_2_, *w*_2_),···,(*p*_*I*_, *w*_*I*_)} by the following formulas.

(17) $$\begin{array}{l} k=\underset{1\leq i\leq I}{arg min}|p_{t}-p_{i}| \end{array}$$

(18) $$\begin{array}{l} \hat{w_{t}}=w_{k} \end{array}$$

The location name $\hat{n}$ can be predicted by randomly selecting a location name from location names in $\hat{w_{t}}$ of the nearest data point.

If word information *w*_*t*_ is observed, then position information $\hat{p_{t}}$ is randomly selected from dataset *D*_*n*_*t*__, which is a set of data *d*_*i*_ = (*p*_*i*_, *w*_*i*_) satisfying the formula *w*_*i*_ ∈ *w*_*t*_. The sampling of position information for each location name was performed 100 times.

#### 4.3.3. Nearest neighbor (vision, position and word)

This method is used only in the name prediction experiment. A datum *d*_*i*_ includes vision information *v*_*i*_, position information *p*_*i*_ = (*x*_*i*_, *y*_*i*_) and word information *w*_*i*_. υ_*i*_ is a value calculated by Formula (1) at a position *p*_*i*_ during training. *w*_*i*_ consists of a set of location names that are obtained at a position *p*_*i*_ during the training. If the vision information *v*_*t*_ and the position information *p*_*t*_ are observed, then the word information $\hat{w_{t}}$ can be calculated with the training dataset *D* = {(υ_1_, *p*_1_, *w*_1_), (υ_2_, *p*_2_, *w*_2_),···,(υ_*I*_, *p*_*I*_, *w*_*I*_)} by using the following formulas.

(19) $$\begin{array}{l} k=\underset{1\leq i\leq I}{arg min}\left(\alpha |v_{t}-v_{i}|+\left(\alpha -1\right)|p_{t}-p_{i}|\right) \end{array}$$

(20) $$\begin{array}{l} \hat{w_{t}}=w_{k} \end{array}$$

where α is the weight coefficient between vision and position information. α was set as 0.3 in the validation dataset empirically. The location name $\hat{n}$ can be predicted by randomly selecting a location name from the location names in $\hat{w_{t}}$ of the nearest data point.

#### 4.3.4. Multimodal HDP

Multimodal HDP (Nakamura et al., 2011) enables the multimodal handling of HDP (Teh et al., 2005), which is a method of categorizing observed data based on a Bayes generative model, in the topic distribution of latent Dirichlet allocation (LDA) as HDP. The graphical model and definition of variables in the multimodal HDP are shown in the Supplementary Material. Here, multimodal HDP was trained using vision, position, and word information. If vision information $w_{t}^{v}$ and position information ${\text{w}}_{t}^{p}$ are observed at a time *t*, then the posterior probability of word information $w_{t}^{w}$ can be calculated by the following formula:

(21) $$\begin{array}{l} p\left(w_{t}^{w}|ẑ,{\text{w}}^{w},{\text{w}}^{\upsilon },{\text{w}}^{p},w_{t}^{\upsilon },w_{t}^{p},\pi ,\eta^{w},\eta^{\upsilon },\eta^{p}\right)=\\ \underset{z_{t}}{\sum }p\left(w_{t}^{w}|z_{t},{ẑ}^{w},ĉ,{\text{w}}^{w},\eta^{w}\right)p\left(z_{t}|{ẑ}^{\upsilon },{ẑ}^{p},{\text{w}}^{\upsilon },{\text{w}}^{p},w_{t}^{\upsilon },w_{t}^{p},\pi ,\eta^{\upsilon },\eta^{p}\right) \end{array}$$

The location name $\hat{n}$ can be predicted by the maximum value of the calculated posterior probability.

If word information $w_{t}^{w}$ is obtained at a time *t*, then a category $z_{t}^{w}$ can be predicted by Formula (22) and selecting position information $\hat{p}$ randomly from dataset $D_{z_{t}^{w}}$, which is a set of position data categorized into $z_{t}^{w}$.

(22) $$\begin{array}{l} z_{t}^{w}\sim p\left(z_{t}^{w}|{\text{z}}_{-t}^{w},w_{t}^{w},{\text{w}}^{w},{\text{w}}^{\upsilon },{\text{w}}^{p},\eta^{w},\eta^{\upsilon },\eta^{p},\pi \right) \end{array}$$

The sampling of position information for each location name was performed 100 times. In the multimodal HDP, the hyper-parameters π, η were set as π = 50, η^υ^ = 5.0 × 10^−1^, η^*p*^ = 1.0 × 10^−1^, η^*w*^ = 1.0 × 10^−1^ in the validation dataset. The category *z* of each data is trained with the hyper-parameters.

#### 4.3.5. Spatial concept formation

Spatial concept formation (SpCoFo)^5^ is a model that integrates name modalities into the spatial region learning model (Ishibushi et al., 2015). The model forms concepts from multimodal information and predicts unobserved information. The graphical model and definition of variables in the spatial concept formation model are shown in the Supplementary Material. The posterior probability of word information $w_{t}^{n}$ after obtaining vision information $w_{t}^{\upsilon }$ and position information *p*_*t*_ was calculated by the following formula:

(23) $$\begin{array}{ll} p\left(w_{t}^{n}|p_{t},w_{t}^{\upsilon }\right)=\underset{z_{t}}{\sum }p\left(w_{t}^{n}|z_{t}\right)p\left(z_{t}|p_{t},w_{t}^{\upsilon }\right)\\ = & \underset{z_{t}}{\sum }p\left(w_{t}^{n}|\beta_{z_{t}}^{n}\right)p\left(p_{t}|\mu_{z_{t}},\Sigma_{z_{t}}\right)p\left(w_{t}^{\upsilon }|\beta_{z_{t}}^{\upsilon }\right) \end{array}$$

The location name $\hat{n}$ can be predicted by the maximum value of the calculated posterior probability.

The prediction of position $\hat{p_{t}}$ after obtaining word information $w_{t}^{n}$ was calculated by estimating a category *z*_*t*_ and sampling position information $\hat{p}$ using the following formulas.

(24) $$\begin{array}{l} z_{t}=\underset{z_{t}}{\mathrm{argmax}}p\left(z_{t}|w_{t}^{n}\right)\\ \hat{p_{t}}\sim p\left(p_{t}|\mu_{z_{t}},\Sigma_{z_{t}}\right) \end{array}$$

The sampling of position information for each location name was performed 100 times. In the spatial concept formation, the hyper-parameters π, η, μ_0_, κ_0_, ψ_0_, and ν_0_ were set as π = 50, η^υ^ = 5.0 × 10^−1^, η^*w*^ = 1.0 × 10^−1^, μ_0_ = (*x*_*center*_, *y*_*center*_), $\kappa_{0}=3.0\times 10^{-2}$, ψ_0_ = *diag*[0.05, 0.05, 0.05, 0.05], and ν_0_ = 15 in the validation dataset, respectively. (*x*_*center*_, *y*_*center*_) indicates the center of the map. The category z of each data is trained with the hyper-parameters.

### 4.4. Experimental results

#### 4.4.1. Hierarchical space categorization

**Figure 8:**
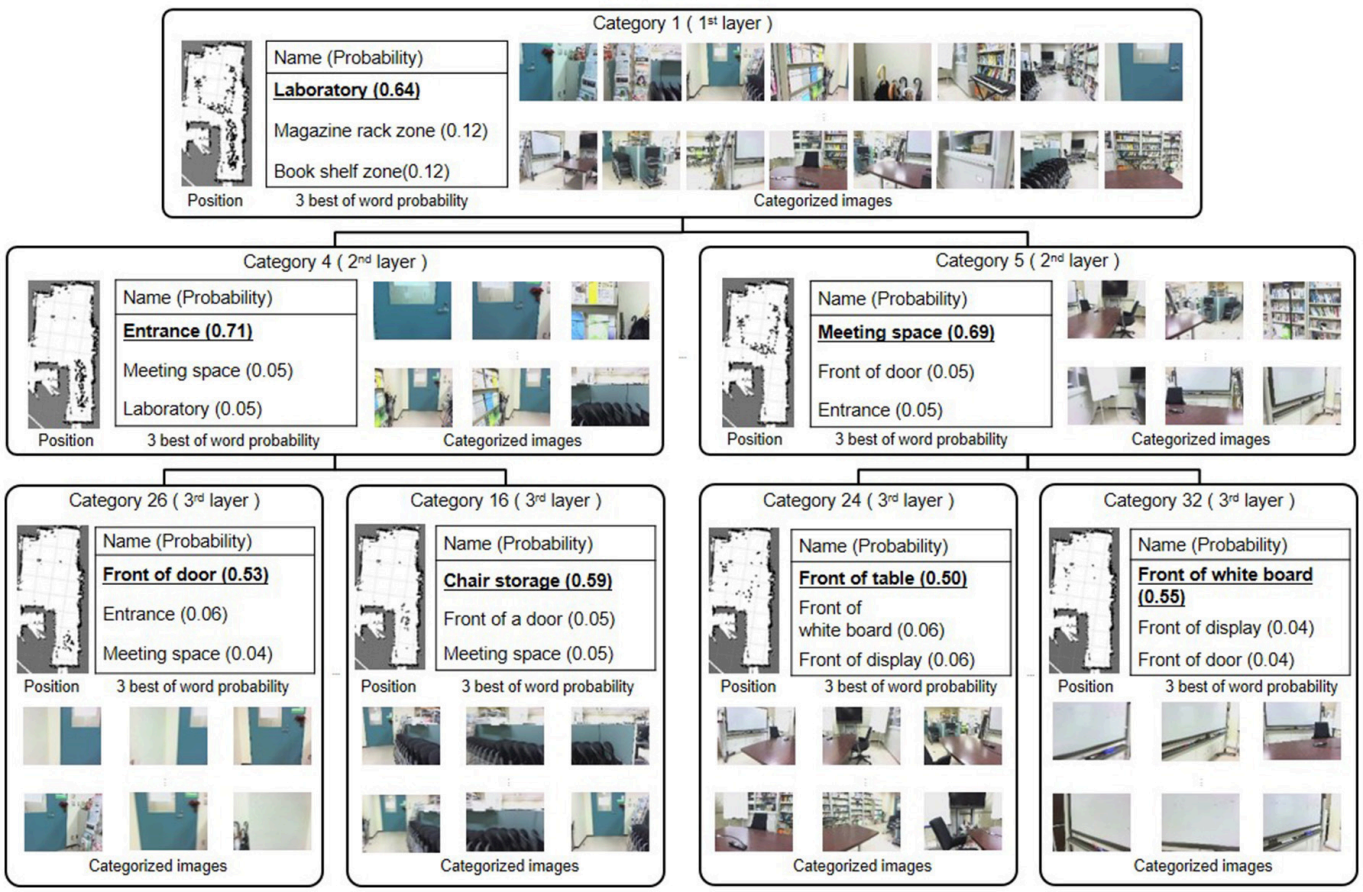
Hierarchical spatial concept formed by the proposed method.

Figure 8 shows some categories formed by the proposed method. Categorized training data at each category are shown by positions, images, and the best three examples from the word probability. The category corresponds to the formed spatial concept. Each category was classified into an appropriate layer in the hierarchy of spatial concepts. One, four, and 28 categories were classified into the 1st, 2nd, and 3rd layers, respectively. The number of categories in each layer was determined by the nCRP based on the model parameter γ, which controls the probability that the data is allocated to a new category.

The 1st layer included only category 1, into which 900 data were allocated. The high-probability word of category 1 was “laboratory,” which referred to the entire experimental environment. Since category 1 contains all the location names, the probabilities for location names becomes relatively low. Nonetheless, the proposed method was able to learn “laboratory,” which was given only about 10% to the training dataset, with high probability compared to the second candidate. In the 2nd layer, 343 data in the vicinity of the entrance in the experimental environment were allocated into category 4. The location name of category 4 with the greatest probability was “entrance.” The 389 data in the region deeper than the entrance in the experimental environment were categorized into category 5, in which “meeting space” had the greatest probability. In the 3rd layer, the data categorized into categories 4 and 5 in the second layer were further, more finely categorized. In categories 26 and 16, which were formed under category 4, “front of the door” and “front of the chair storage” had the greatest probabilities, respectively. 53 and 81 data were allocated into categories 26 and 16, respectively. Position and image data corresponding to “front of the door” and “front of the chair storage” were finely allocated. These results demonstrated that the proposed method can form not only categories in a lower layer such as “front of the chair storage” and “front of the door” but also categories at higher layers such as “entrance” and “laboratory,” and can form its inclusion relations as a hierarchical structure.

#### 4.4.2. Evaluation of categorization

To evaluate the effectiveness of multimodal information on hierarchical space categorization, we compared the categorization results of using the proposed method and those obtained using hLDA, which is a hierarchical categorization method with single modality, i.e., based only on word information. Although the number of layers in ground truth in this experiment is 3, robots can not know the number of hierarchies of the spatial concepts in advance. Therefore, in the proposed method and hLDA, categorization was performed with the number of layers changed from 2 to 5. The accuracy of space categorization was evaluated by calculating mutual information between the ground truth, which consisted of a location name given by humans, and the estimated name, which was the best item in the word probability at a category allocated by the proposed method or by hLDA. Mutual information *I*(*E*; *G*) between estimated name *E* and ground truth *G* in each layer *i* and *j* was calculated by the following formula:

(25) $$\begin{array}{l} I\left(E;G\right)=\underset{g_{j}\in G}{\sum }\underset{e_{i}\in E}{\sum }p\left(e_{i},g_{j}\right)\log\frac{p\left(e_{i},g_{j}\right)}{p\left(e_{i}\right)p\left(g_{j}\right)}. \end{array}$$

When the mutual information become high, the dependency of *e*_*i*_ and *g*_*j*_ can be regarded as high. By using mutual information, accuracy of categorization can be evaluated when the number of layers on ground truth and estimation result is different. Table 4 shows the mutual information for categorization results between hLDA with word information and the proposed method with vision, position, and word information in the training data set. The effectiveness of multimodal information in space categorization was clarified, since the proposed method had a high level of mutual information in all layers. In addition, mutual information was maximized when using the same hierarchical number as in the ground truth. In the subsequent evaluations, the number of layers of the proposed method is set to 3.

**Table 4:** Mutual information for categorization of location names when changing the number of layers in hLDA with word information and the proposed method with vision, position, and word information.

**Method**	**Modality**	**2 layers**	**3 layers**	**4 layers**	**5 layers**
hLDA	Word	0.87	0.71	0.44	0.41
Proposed method	Vision, position, and word	**0.97**	**1.28**	**0.94**	**0.89**

*Mutual information was calculated by Formula 25. Underlined and bold values mean the maximum value in the experimental parameter*.

#### 4.4.3. Evaluation of name prediction and position category prediction

We conducted experiments to verify whether or not the proposed method could form hierarchical spatial concepts, which enable a robot to predict location names and position categories similar to predictions made by humans. In the experiment, (1) the influence of multimodal information on the formation of a hierarchical spatial concept was evaluated by comparing the space-categorization results obtained using the proposed method and using hLDA, which is a hierarchical categorization method with single modality; (2) the similarity between the hierarchical spatial concepts formed by the proposed method and those of humans was evaluated in predicting location names and position categories. The evaluation experiments were performed by cross verification with three data sets that consist of 900 training data and 100 test data with ground truth. The experimental results are indicated by the mean and standard deviation in the three data sets.

To verify whether or not the proposed method can form hierarchical spatial concepts, accuracy evaluation of name prediction and position category prediction through spatial concept use was performed. In the evaluation of name prediction, vision, position, and word information were given to the robot at the training data points. In the test data points, only vision and position information were given. Therefore, the robot has to predict the unobserved word information from the observed vision and position information. Table 5 shows the accuracy of name prediction using the baseline methods, the proposed method, and those made by humans. The most frequent class, nearest neighbor (position and word), nearest neighbor (vision, position, and word), multimodal HDP, and spatial concept formation model were used as the baseline methods. The accuracy of name prediction was calculated by Formula (13) at global, intermediate, and local layers in ground truth. The proposed method and humans predicted location names in three layers. The results of humans consisted of the average accuracy of three subjects familiar with the experimental environment.

**Table 5:** Accuracy of name prediction using the baseline methods, the proposed method, and those made by humans; the accuracy was calculated by using Formula (13).

			**Mean (s.d.)**		
**Method**	**Modality**	**Layer**	**Global**	**Intermediate**	**Local**
Most frequent class	Position and word		**1.00 (0.00)**	0.18 (0.32)	0.09 (0.02)
Nearest neighbor	Position and word		0.12 (0.01)	0.24 (0.02)	0.20 (0.03)
Nearest neighbor	Vision, position and word		0.18 (0.03)	0.28 (0.04)	0.31 (0.04)
Multimodal HDP	Vision, position, and word		0.13 (0.02)	0.54 (0.06)	0.24 (0.07)
SpCoFo	Vision, position, and word		0.25 (0.13)	0.23 (0.15)	0.36 (0.13)
		1st	**1.00 (0.00)**	0.00 (0.00)	0.00 (0.00)
Proposed method	Vision, position, and word	2nd	0.00 (0.00)	**0.96 (0.04)**	0.01 (0.02)
		3rd	0.00 (0.00)	0.04 (0.04)	**0.55 (0.07)**
		1st	1.00 (0.00)	0.00 (0.00)	0.00 (0.00)
Humans		2nd	0.00 (0.00)	0.98 (0.02)	0.00 (0.00)
		3rd	0.00 (0.00)	0.03 (0.04)	0.74 (0.10)

*The accuracy is indicated by the mean and standard deviation (s.d.). Underlined and bold values mean the maximum value in the experimental parameter*.

Compared with the accuracy obtained using the baseline methods, higher accuracies were obtained by the proposed method in the 1st, 2nd, and 3rd layers. It was assumed that weak features buried in the lower layer in the baseline methods were categorized as features of the higher layer in the proposed method. The proposed method enabled a robot to predict location names close to predictions made by humans by selecting the appropriate layer depending on the situation.

Table 6 shows the evaluation results of position category prediction using the baseline methods, the proposed method, and those made by humans. In the evaluation, the most frequent class, nearest neighbor (position and word), multimodal HDP, and spatial concept formation model were used as the baseline methods. The position category prediction was evaluated in terms of precision, recall, and F-measure, which were calculated by Formula (14).

**Table 6:** Precision, recall, and F-measure evaluation of position category prediction using the baseline methods, the proposed method, and those made by humans in global, intermediate, and local; the precision, recall, and F-measure were calculated by using Formula (14).

**Method**	**Precision**			**Recall**			**F-measure**		
	**Global**	**Intermediate**	**Local**	**Global**	**Intermediate**	**Local**	**Global**	**Intermediate**	**Local**
Most frequent class	**1.00 (0.01)**	0.49 (0.01)	0.37 (0.03)	0.12 (0.02)	0.17 (0.02)	0.15 (0.03)	0.22 (0.03)	0.25 (0.02)	0.20 (0.02)
Nearest neighbor	**1.00 (0.00)**	0.93 (0.03)	**0.67 (0.03)**	0.12 (0.03)	0.26 (0.04)	0.23 (0.04)	0.22 (0.04)	0.41 (0.05)	0.33 (0.03)
Multimodal HDP	**1.00 (0.00)**	0.95 (0.02)	0.53 (0.03)	0.12 (0.01)	0.26 (0.04)	0.26 (0.02)	0.21 (0.02)	0.40 (0.05)	0.33 (0.02)
SpCoFo	0.82 (0.00)	0.62 (0.04)	0.35 (0.04)	0.16 (0.01)	0.32 (0.02)	**0.38 (0.04)**	0.27 (0.02)	0.42 (0.01)	0.35 (0.04)
Proposed method	**1.00 (0.00)**	**0.96 (0.03)**	0.59 (0.05)	**0.18 (0.01)**	**0.34 (0.02)**	0.36 (0.04)	**0.30 (0.02)**	**0.50 (0.02)**	**0.43 (0.01**)
Humans	1.00	0.99	0.76	0.19	0.50	0.49	0.32	0.65	0.56

*In the experiment, the modalities of the nearest neighbor were position and word. The results are indicated by the mean and standard deviation as mean (s.d.). Underlined and bold values mean the maximum value in the experimental parameter*.

Compared with results obtained by the baseline methods, higher values of precision and recall were obtained by the proposed method in the global and intermediate layers. In the local layer, higher values of precision and recall were obtained by Nearest neighbor and Spatial Concept Formation (SpCoFo), respectively. However, in the F-measure, which is a harmonic mean between precision and recall, the proposed method has the largest values in the global, intermediate, and local layers. The reason why the recall and F-measure values were lower than the precision is that only 100 data points were predicted and plotted for regions with 100 grids or more, as shown in Figure 7. In the result of F-measure, independent *t*-tests were performed in nine samples consisting of three data sets with three types of ground truth: global, intermediate, and local. In the proposed method, the *p*-values of the Most frequent class, Nearest neighbor, multimodal HDP, and SpCoFo were 0.00012, 0.00004, 0.00003, and 0.00051, respectively, and significant differences were observed with (*p* < 0.05). As the reason why the result of humans were not perfect, some errors were found in the boundary of the place. For example, the boundary between “Book shelf zone” and “front of the table,” and the edge of the region called “front of the door” were different depending on the human. The centricity of the place is consistent, but the region includes ambiguity even among humans. The experimental results show that the proposed method enabled a robot to predict position categories closer to predictions made by humans than possible using the baseline methods.

In the experiments for location name and position category prediction, the proposed method showed higher performance than the baseline methods. In the baseline methods, i.e., multimodal HDP and SpCoFo, since the feature space is classified uniformly, the location concepts are formed non-hierarchically. For example, an upper concept, e.g., meeting space, is embedded in the lower concepts, e.g., front of the table and front of the display. Therefore, the place called “Meeting space” is learned as a place different from the places called “front of the table” and “front of the display.” Since the proposed method forms concepts by extracting the similarity of knowledge in the upper concept, it is possible to form an upper concept without interfering with the formation of the lower concept. For this reason, the proposed method was able to show high performance in the experiments of name and position category prediction with global, intermediate, and local.

In human-robot interactions in home environments, location names as word information are given to only a part of the training data from a user. We evaluated the robustness of the proposed method in terms of the naming rate in order to verify how name and position category prediction performance changes with decreasing naming rate. In this experiment, the formation of spatial concepts using the proposed method was performed using the training data with the naming rate changed to 1, 2, 5, 10, and 20% successively. The naming rates of 1 or 20% mean that 9 or 180 of the 900 training data contained location names, while the remaining data did not contain any location name. Table 7 shows the accuracy of name prediction and the F-measure of position category prediction for each naming rate. In the results of name prediction and position category prediction, it was confirmed that learning progresses in the global layer earlier than in the intermediate and local layers. It was clarified that overall prediction ability did not decrease greatly owing to the decreased naming rate, but gradually decreased from the lower layer. In this experiment, we performed spatial concept formation without prior knowledge in only one environment, but it is possible to increase learning efficiency by giving parameters of models estimated in other environments as prior probabilities. The transfer learning of spatial concepts will be performed in the future.

**Table 7:** Robustness evaluation of the proposed method with respect to naming rate: accuracy in name prediction indicates the maximum value of the three layers.

**Naming rate**	**Name prediction (accuracy)**			**Position prediction (F-measure)**		
	**Global**	**Intermediate**	**Local**	**Global**	**Intermediate**	**Local**
1%	1.00	0.68	0.14	0.29	0.46	0.30
2%	1.00	0.77	0.26	0.29	0.47	0.31
5%	1.00	0.92	0.35	0.28	0.36	0.37
10%	1.00	0.92	0.58	0.30	0.46	0.37
20%	1.00	0.92	0.63	0.31	0.50	0.44
Humans	1.00	0.96	0.76	0.32	0.65	0.56

## 5. Application examples for human support robots

Application examples of the hierarchical spatial concept using the proposed method are demonstrated in this section. We implemented the proposed method for the Toyota human support robot (HSR)^6^ and created application examples in which the robot moves based on human linguistic instructions and describes its self-position linguistically in an experimental field assuming a home environment.

**Figure 9:**
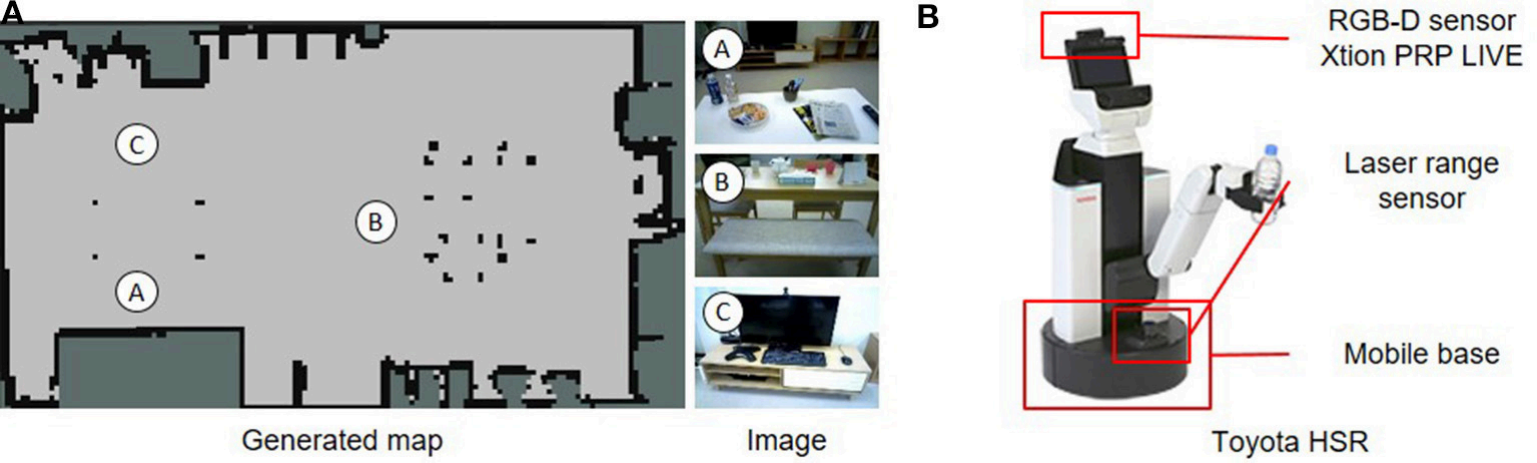
Experimental environment and robot for demonstrating application examples. **(A)** Environments A, B, and C in the generated map show positions in which images were captured. **(B)** Human support robot produced by the Toyota Company.

The home environment and the robot used are shown in Figure 9. There were two tables as shown in Figure 9A, A and B. In the environment, whether the robot could move based on linguistic instructions including the hierarchical structure of spaces such as “front of the table in the living room” and “front of the table in the dining room” was verified. In Figure 9B, an RGB-D sensor and a laser range sensor were used to capture images and to estimate self-position, respectively. The packages^7^: hector_slam and omni_base for mapping, localization, and moving were used with ROS Indigo^8^ to navigate the robot to the predicated position.

The robot collected 715 training data consisting of images, positions, and word information and formed a hierarchical spatial concept using the proposed method. Location names were given to 20% of total training data. Rospeex (Sugiura and Zettsu, 2015) was used to recognize human speech instructions and convert them into text information. In the experiment, the dimensions of the information vectors *w*^υ^, *w*^*p*^, and *w*^*w*^ were 1,000, 64, and 16, respectively.

**Figure 10:**
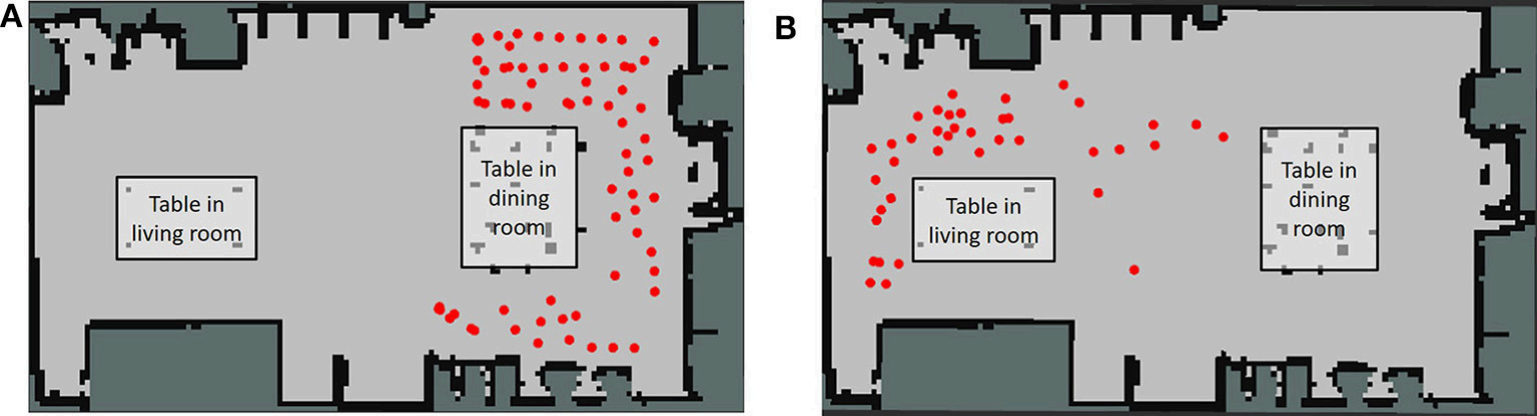
Position category prediction using a hierarchical structure based on linguistic instructions from the user. **(A)** Positions for the front of the table in the living room. **(B)** Positions for the front of the table in the dining room.

The two places predicted by Formula (12) based on the speech instructions, i.e., “go to the front of the table in the living room” and “go to the front of the table in the dining room” are shown in Figures 10A,B, respectively. Predicted position categories indicated by red dots show that the “front of the table in the living room” and the “front of the table in the dining room” were recognized as different places using the space concept in the higher layer.

**Figure 11:**
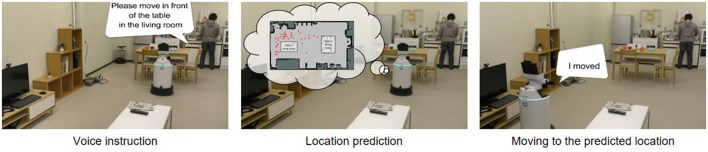
Movement based on speech instructions from the user through the hierarchical spatial concept.

**Figure 12:**
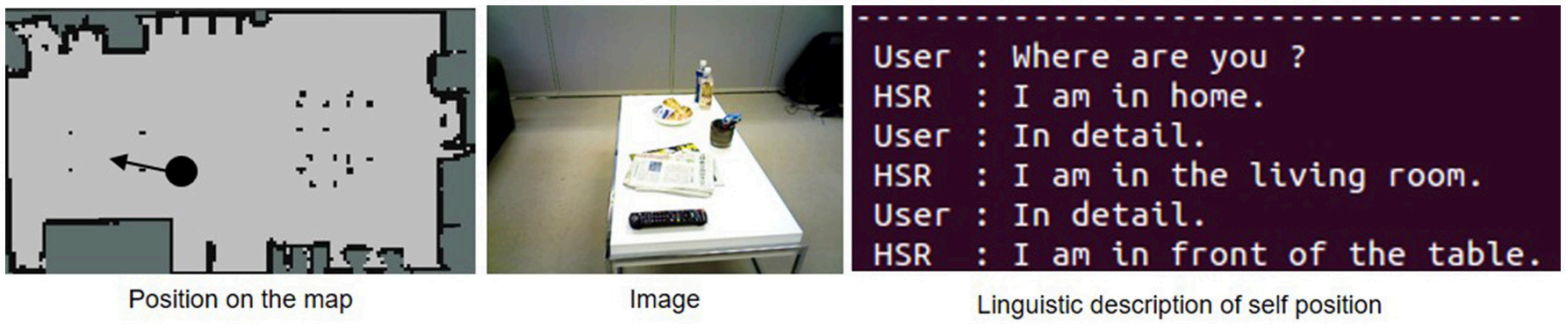
Linguistic description of self-position based on communication between the user and the robot using the hierarchical spatial concept.

Figure 11 shows how the robot moved based on human speech instructions in the experiment. The robot recognized human speech instructions using rospeex and predicted position categories with the Formula (12) using a hierarchical spatial concept. It moved to the instructed place by sampling randomly from the predicted positions. Figure 12 shows an application example in which the robot described its self-position linguistically. The robot observed its self-position and image and predicted the name of its self-position by calculating Formula 11 using the hierarchical spatial concept. As shown in the left side of Figure 12, the proposed method enabled the robot to describe its self-position linguistically with different layers. We demonstrated application examples using the formed hierarchical spatial concept in the service scene in a home environment. The movie of the demonstration and training dataset can be found at the URL^9^.

## 6. Conclusions

We assumed that a computational model that considers the hierarchical structure of space enables robots to predict the name and position of a space close to the corresponding prediction by humans. In our assumptions, we proposed a hierarchical spatial concept formation method based on a Bayesian generative model with multimodal information, i.e., vision, position, and word information, and developed a robot that can predict unobserved location names and position categories based on observed information using the formed hierarchical spatial concept. We conducted experiments to form a hierarchical spatial concept using a robot and evaluated its ability in name prediction and position category prediction.

The experimental results for name and position category prediction demonstrated that, relative to baseline methods, the proposed method enabled the robot to predict location names and position categories closer to predictions made by humans. Application examples using the hierarchical spatial concept in a home environment demonstrated that a robot could move to an instructed place based on human speech instructions and describe its self-position linguistically through the formed hierarchical spatial concept. The experimental results and application example demonstrated that the proposed method enabled the robot to form spatial concepts in abstract layers and use the concepts for human-robot communications in a home environment. This study showed that it the name and position of a location could be predicted, even in a home, using generalized spatial concepts. Furthermore, by conducting additional learning in each house, a spatial concept adapted to the environment can be formed.

The limitation of this study is as follows. In the feature extraction of the position information, hierarchical k-means method was utilized to convert the position information (*x, y*) into the position histogram. In the experiment, 389 and 511 data were allocated to two clusters at the top layer *c*_1_. In the bottom layer *c*_6_, the number and standard deviation of the data allocated to each of the 64 clusters were 14.1 and 12.2, respectively. There is some bias between the clusters. The hierarchical k-means makes it possible to convert the position information into the position histogram including hierarchical spatial features. However, nearby data points at a classification boundary, which are classified into different clusters on a high level, are regarded as very different. We are considering a method to reduce bias in space while maintaining hierarchical features of space. As for the number of location names, at section 4 and 5 in the experiments, the numbers of location names were 15 and 16, respectively. The number of location names increases with increase in the numbers of teachings and users. If the robot learns the location names from several users over a long term, an algorithm to remove location names with low probability of observation is needed in order to improve the learning efficiency.

As future work, we will generalize the spatial concepts for various environments and perform transition learning of spatial concepts with the generalized spatial concepts as prior knowledge.

## Author contributions

YH designed the study, and wrote the initial draft of the manuscript. HK and MI contributed to analysis and interpretation of data, and assisted in the preparation of the manuscript. TT has contributed to data collection and interpretation, and critically reviewed the manuscript. All authors approved the final version of the manuscript, and agree to be accountable for all aspects of the work in ensuring that questions related to the accuracy or integrity of any part of the work are appropriately investigated and resolved.

### Conflict of interest statement

The authors declare that the research was conducted in the absence of any commercial or financial relationships that could be construed as a potential conflict of interest.

## Footnotes

^1^GPSR Command Generator: https://github.com/kyordhel/GPSRCmdGen

^2^ILSVRC2013: http://www.image-net.org/challenges/LSVRC/2013/

^3^Navigation Stack: http://wiki.ros.org/navigation

^4^ROS Hydro: http://wiki.ros.org/hydro

^5^Spatial Concept Formation: https://github.com/EmergentSystemLabStudent/Spatial_Concept_Formation

^6^Toyota Global Site—Partner Robot Family: http://www.toyota-global.com/innovation/partner_robot/family_2.html

^7^hector_slam: http://wiki.ros.org/hector_slam

^8^ROS Indigo: http://wiki.ros.org/indigo

^9^Multimedia - emlab page: https://emlab.jimdo.com/multimedia/
