# Supplementary material for: Hierarchical Spatial Concept Formation Based on Multimodal Information for Human Support Robots
Source: Front Neurorobot. 2018 Mar 13;12:11. doi: 10.3389/fnbot.2018.00011 (PMC5859180; doi:10.3389/fnbot.2018.00011)
Supplement: Supplementary file 1 [file Presentation1.PDF]

Supplementary Material: Hierarchical Spatial Concept Formation Based on Multimodal Information for Human Support Robots

Yoshinobu Hagiwara*, Masakazu Inoue, Hiroyoshi Kobayashi and Tadahiro Taniguchi

*Correspondence:
Yoshinobu Hagiwara
yhagiwara@em.ci.ritsumei.ac.jp

1 SUPPLEMENTARY TABLES AND FIGURES

The graphical models of multimodal HDP and spatial concept formation used as a baseline method in experiments are shown in the respective figures, respectively. Variable definitions of the graphical models of Figure S1a and S1b are shown in Table S1 and S2.

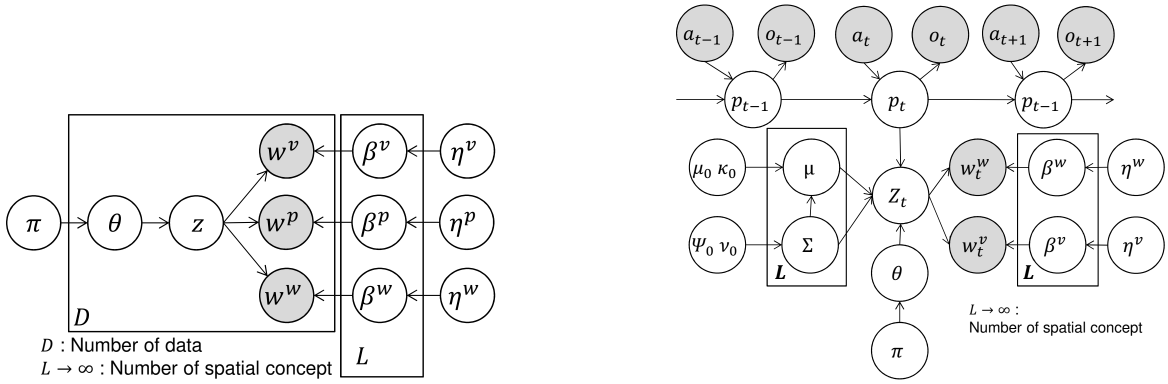

(a) multimodal HDP

(b) Spatial concept formation

Figure S1: Graphical models for baseline methods, i.e. multimodal HDP and spatial concept formation

Table S1. Definition of variables in multimodal HDP

z	Index of category
w^v, w^p, w^w	vision, position and word information
$\beta^v, \beta^p, \beta^w$	Parameters for multinomial distribution of w^v, w^p, w^w
η^v, η^p, η^w	Parameters of Dirichlet prior distribution of $\beta^v, \beta^p, \beta^w$
θ	Parameters for multinomial distribution of z
π	Hyper parameter of θ

Table S2. Definition of variables in Spatial concept formation

z	Index of category
a_t, o_t, p_t	action, distance and position information
μ, Σ	Parameters for Gaussian distribution
μ_0, κ_0 Ψ_0, ν_0	Parameters for Gauss Wishart distribution
w^v, w^w	vision and word information
β^v, β^w	Parameters for multinomial distribution of w^v, w^w
η^v, η^w	Parameters of Dirichlet prior distribution of β^v, β^w
θ	Parameters for multinomial distribution of z
π	Hyper parameter of θ
